# Drug-coated balloon angioplasty with provisional stenting versus primary stenting for the treatment of de novo coronary artery lesions: REC-CAGEFREE I trial rationale and design

**DOI:** 10.1186/s12872-024-03974-0

**Published:** 2024-06-24

**Authors:** Chao Gao, Xingqiang He, Yunpeng Liu, Jianzheng Liu, Zhiwei Jiang, Bin Zhu, Xing Qin, Yunlong Xia, Tingting Zhang, Ping Wang, Ruining Zhang, Yoshinobu Onuma, Jielai Xia, Duolao Wang, Patrick Serruys, Ling Tao

**Affiliations:** 1 Department of Cardiology, Xijing Hospital, Xi’an, 710032 China; 2 Department of Statistics, Air Force Medical University, Xi’an, 710000 China; 3 Department of Cardiology, University of Galway, Galway, H91 TK33 Ireland; 4 Biostatistics Unit, Liverpool School of Tropical Medicine, Liverpool, L3 5QA UK

**Keywords:** Drug-coated balloon, Drug-eluting stent, De novo lesions, Coronary artery disease

## Abstract

**Background:**

Percutaneous coronary intervention (PCI) with primary stenting, which stands for stent implantation regardless of obtaining satisfactory results with balloon angioplasty, has superseded conventional plain old balloon angioplasty with provisional stenting. With drug-coated balloon (DCB), primary DCB angioplasty with provisional stenting has shown non-inferiority to primary stenting for *de novo* coronary small vessel disease. However, the long-term efficacy and safety of such a strategy to the primary stenting on clinical endpoints in *de novo* lesions without vessel diameter restrictions remain uncertain.

**Study design:**

The REC-CAGEFREE I is an investigator-initiated, multicenter, randomized, open-label trial aimed to enroll 2270 patients with acute or chronic coronary syndrome from 43 interventional cardiology centers in China to evaluate the non-inferiority of primary paclitaxel-coated balloons angioplasty to primary stenting for the treatment of *de novo*, non-complex lesions without vessel diameter restrictions. Patients who fulfill all the inclusion and exclusion criteria and have achieved a successful lesion pre-dilatation will be randomly assigned to the two arms in a 1:1 ratio. Protocol-guided DCB angioplasty and bailout stenting after unsatisfactory angioplasty are mandatory in the primary DCB angioplasty group. The second-generation sirolimus-eluting stent will be used as a bailout stent in the primary DCB angioplasty group and the treatment device in the primary stenting group. The primary endpoint is the incidence of Device-oriented Composite Endpoint (DoCE) within 24 months after randomization, including cardiac death, target vessel myocardial infarction, and clinically and physiologically indicated target lesion revascularization.

**Discussion:**

The ongoing REC-CAGEFREE I trial is the first randomized trial with a clinical endpoint to assess the efficacy and safety of primary DCB angioplasty for the treatment of *de novo*, non-complex lesions without vessel diameter restrictions. If non-inferiority is shown, PCI with primary DCB angioplasty could be an alternative treatment option to primary stenting.

**Trial registration:**

Registered on clinicaltrial.gov (NCT04561739).

**Supplementary Information:**

The online version contains supplementary material available at 10.1186/s12872-024-03974-0.

## Introduction

Since the bare-metal stent era, primary plain old balloon angioplasty (POBA) with provisional stenting has been superseded by routine coronary stenting for the treatment of *de novo* coronary lesions because POBA had a higher risk of repeat revascularization [1]. This notion was maintained in the drug-eluting stents (DES) era, even for patients who require urgent non-cardiac surgery or high bleeding risks, as short-duration dual antiplatelet therapy may be reasonable with both strategies [2–4]. However, stent implantation continues to face notable challenges as there is a permanent metallic scaffold left behind in the vessel. Stents may distort and constrain the coronary vessel, limit vessel pulsatility, and adaptive remodeling [5], and promote chronic inflammation, which in turn increases the risk of late stent thrombosis and restenosis by approximately 2% per year [2].

Drug-coated balloons (DCB) represent a contemporary therapeutic effort in the treatment of coronary artery disease (CAD) [6]. Upon reaching the target lesion, the expansion of DCB can rapidly deliver antiproliferative drugs into the arterial wall through a lipophilic matrix during angioplasty without the necessity of implanting a scaffold [7]. This feature of DCB has the potential to minimize the negative effects associated with stent-related maladaptive biologic response [8]. Currently, the management of in-stent restenosis (ISR) by DCB is considered a Class IA recommendation [2]. The safety and effectiveness of the DCB strategy have also been demonstrated in *de novo* small vessels [9], acute coronary syndromes [10, 11], and high-bleeding risk patients [12]. Moreover, the application of DCB is gradually expanding to include all *de novo* coronary arteries without diameter restrictions. However, the use of DCB in such cases is still controversial [13, 14] due to the lack of randomized study with powered clinical endpoints.

To fill the knowledge gap, we designed the REC-CAGEFREE I trial to revive the longstanding debate between angioplasty and stenting in contemporary settings. The trial will investigate the potential non-inferiority of the primary paclitaxel-coated balloon angioplasty with provisional stenting compared to the primary second‐generation sirolimus-eluting stenting for the treatment of *de novo* coronary lesions without vessel diameter restrictions. The evaluation will be conducted through a randomized controlled trial, focusing on a composite clinical endpoint comprising cardiac death, target vessel myocardial infarction, and clinically indicated target lesion revascularization at the 24-month follow-up.

## Study design

### Objectives and hypothesis

**Fig. 1:**
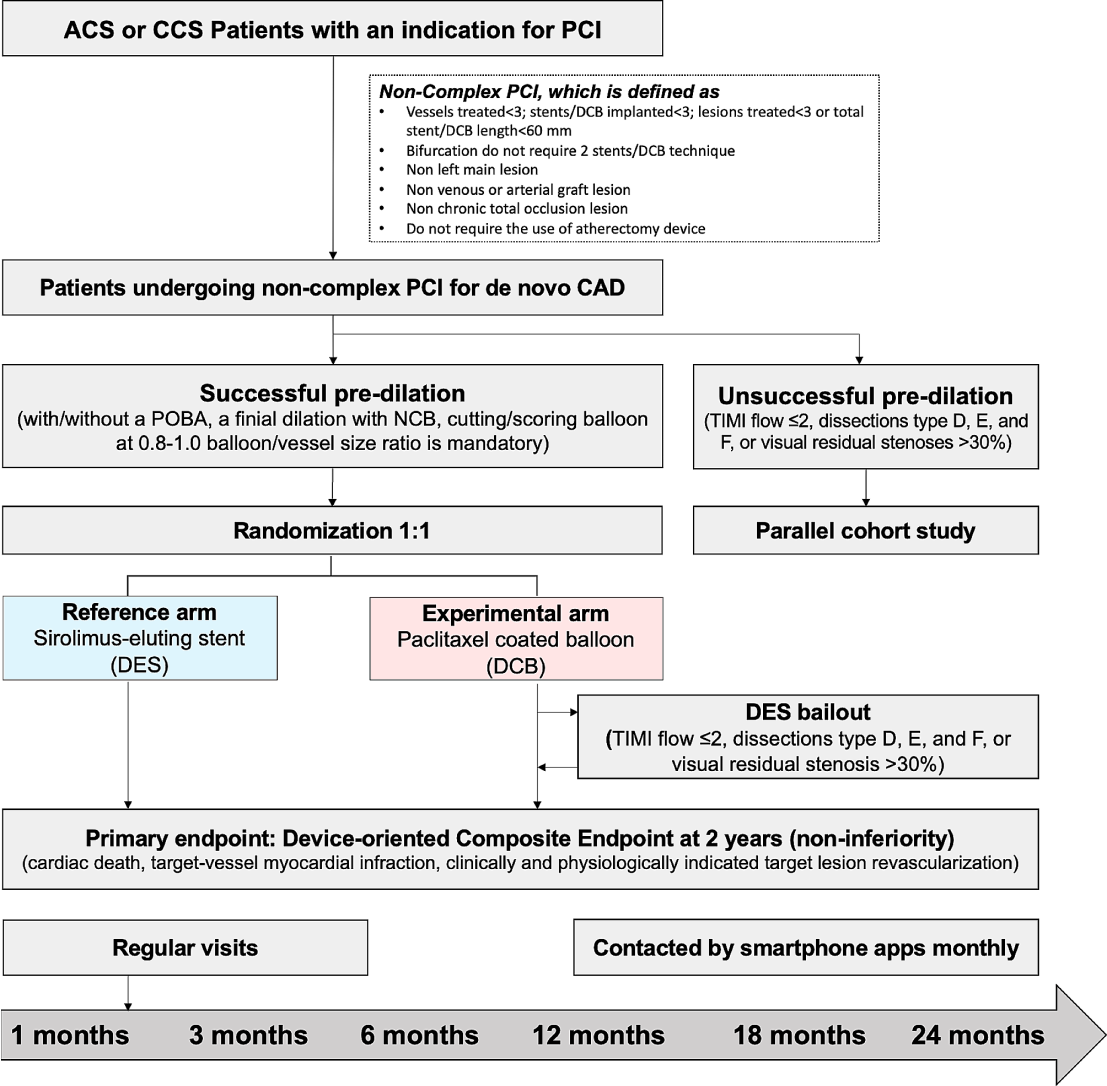
Study flow chart. CAG, Coronary Angiography; PCI, percutaneous coronary intervention; CAD, coronary artery disease; TIMI, Thrombolysis In Myocardial Infarction; POBA, plain old balloon angioplasty; NCB, non-compliant balloon; DoCE, Device-oriented Composite Endpoint; TV-MI, target vessel myocardial infarction; CPI-TLR, clinically and physiologically indicated target lesion revascularization

The REC-CAGEFREE I trial (ClinicalTrials.gov, NCT04561739) is an investigator-initiated, multicenter, prospective, randomized, open-label trial aimed to enroll 2270 patients from ≥ 40 interventional cardiology centers in China. The primary objective of the trial is to test the non-inferiority of the primary balloon angioplasty strategy with paclitaxel-coated balloons (Experimental arm) to the primary stenting strategy with second‐generation sirolimus-eluting stents (Reference group) for the treatment of *de novo* lesions without vessel diameter restrictions in the setting of non-complex percutaneous coronary intervention (PCI). The incidence of Device-oriented Composite Endpoint (DoCE) at 24 months will be assessed as the primary endpoint (Fig. 1).

### Study organization and funding

This trial is investigator-initiated and obtained grant support from Xijing Hospital (Xi’an, China; Grant No. XJZT24LY36) and unrestricted grant support from Shenqi Medical (Shanghai, China) and Microport Medical Group (Shanghai, China). Apart from this sponsorship, Shenqi and Microport will not be involved in the design, execution, or decision to publish the study. The steering committee has a pivotal role with overall responsibility for the concept, design, and execution of the study progress in accordance with scientific, medical, ethical, and practical elements. The committee will convene a series of meetings to ensure the effective management and execution of the study, including data acquisition, quality control, security, analysis, and reporting. The study will follow the ethical principles outlined in the Declaration of Helsinki and has received approval for its protocol from the institutional review board at each participating center.

### Study population

The complete inclusion and exclusion criteria are shown in Table 1. Patients indicated for PCI either due to acute (including STEMI, NSTEMI, and unstable angina) or chronic coronary syndrome are eligible [15, 16]. To be considered suitable for enrollment, the target lesion must be *de novo*, non-complex, and successfully pre-dilatated. Therefore, patients will be consented before the angiography but are formally included and randomized if it is confirmed that all angiographic criteria are met and the target lesion has been successfully pre-dilatated (Fig. 1). To ensure that eligible patients fully comprehend the purpose and procedures of the investigation without encountering any language barriers, the study may opt to enroll patients of Chinese nationality and ethnicity exclusively. To ensure adherence to the study protocol, a training course was organized at each center, led by TL and CG, to ensure that the investigators comprehended and followed the protocol effectively. The eligibility review committees of each participating site, comprising of TL, CG, and the investigators of each site, will conduct a thorough online assessment of cases being screened at the site (after the completion of pre-dilatation) to ensure that all enrolled participants have fulfilled the angiographic and lesion preparation criteria.

**Table 1:** Inclusion and Exclusion criteria

**Inclusion criteria**	
1. Indicated for PCI either due to acute or chronic coronary syndrome	
2. Patients with *de novo*, non-complex lesion^*^ and underwent successful pre-dilatation^**^	
3. Able to complete the follow-up and compliant with the prescribed medication	
*Non-complex PCI is defined as meeting all the following criteria: *1) Planned numbers of lesions/vessel to be treated < 3, planned DES/DCB implanted < 3, or planned total DES/DCB length ≤ 60 mm* *2) Bifurcation does not require 2 DES/DCB* *3) Non left main lesion* *4) Non venous or arterial graft lesion* *5) Non chronic total occlusion lesion* *6) Do not require the use of an atherectomy device* **Successful pre-dilatation is defined as fulfilling all the following criteria: *1) Achieving Thrombolysis In Myocardial Infarction (TIMI) flow grade 3* *2) Without National, Heart, Lung, and Blood Institute (NHLBI) classification defined dissections type D, E, and F* *3) Residual stenosis < 30% after balloon pre-dilation (visual assessment)* *4) Without serious complications requiring the termination of PCI*	
**Exclusion criteria**	
1. Under the age of 18	
2. Unable to provide informed consent	
3. Patient is a woman who is pregnant or nursing	
4. Known contraindication to medications such as Aspirin, Heparin, antiplatelet drugs, or contrast	
5. Currently participating in another trial and not yet at its primary endpoint	
6. Concurrent medical condition with a life expectancy of less than 2 years	
7. Previous intracranial haemorrhage	
8. In stent stenosis requiring revascularization (defined as stenosis ≥ 50% by visual or positive functional assessments in any vessel)	
9. Atrial fibrillation with OAC	
10. Prior CABG	
11. Cardiogenic shock	

### Randomization

Eligible patients who have signed the informed consent will be randomized at a 1:1 ratio using web-based software to be assigned to either the primary DCB angioplasty or primary stenting group. Web-response dynamic-block randomization, utilizing varying blocks of 2, 4, or 6, will allocate random assignment stratified by center.

Patients who fulfill the angiographic criteria (de novo and non-complex lesions) but have unsuccessful pre-dilatation will not be randomized. These patients will be included in a separate parallel cohort study (Fig. 1). The cohort study will be implemented only in sites that agree to join. PCI with DES is recommended for these patients.

### Study procedures

Investigators may exercise discretion in utilizing antithrombotic medications, glycoprotein IIb/IIIa inhibitors, intravascular imaging, or fractional flow reserve. Complete revascularization in one PCI session is recommended. If a staged procedure becomes necessary, it will be documented during the index procedure, and the patient shall use the same allocated strategy and revascularized within 45 days post-index procedure. Any revascularization that is unplanned or beyond the indicated period will be considered a potential event and adjudicated by the independent clinical-event adjudication committee (CEC).

#### Selecting de novo, non-complex lesion and lesion pre-dilatation

As aforementioned, to be considered suitable for enrollment, the target lesion must be *de novo*, non-complex, and successfully pre-dilatated. Non-complex lesion is defined as fulfilling all of the following criteria [17]: (1) Planned numbers of lesions/vessel to be treated < 3, planned DES/DCB implanted < 3, or planned total DES/DCB length < 60 mm; (2) Bifurcation does not require two DES/DCB; (3) Non-left main lesion; (4) Non-venous or arterial graft lesion; (5) Non-chronic total occlusion lesion; (6) Do not require the use of an atherectomy device.

Optimized and successful pre-dilatation includes the requirement of with or without a plain old balloon angioplasty (POBA), a pre-dilation prior to DCB angioplasty shall be performed with a non-compliant balloon, cutting balloon, or scoring balloon at 0.8-1.0 balloon/vessel size ratio. After lesion preparation, a 10-minute observational period should be conducted, followed by an angiogram to ensure satisfactory lesion preparation, which consists of the following criteria: 1) ≤ 30% residual stenosis (visual); 2) Thrombolysis In Myocardial Infarction (TIMI) flow grade 3; 3) the absence of a dissection type D, E, and F according to NHLBI classification; and 4) without serious complications. If the pre-dilatation is deemed unsuccessful, patients will be disqualified from entering the randomization.

#### Randomization to the primary DCB angioplasty strategy

The performance of DCB angioplasty adheres to the recommendations of the German Consensus Group on DCB interventions [18] and the Third Report of the International DCB Consensus Group with adjustments [19]. The DCB angioplasty should only be used after successful pre-dilatation. Subsequently, the DCB, on each side longer than the DCB by at least 2–3 mm (visual) to avoid geographical mismatch, is inflated at nominal pressure for 30–45 s. Similarly, a 10-minute observational period should be conducted, followed by an angiogram to ensure satisfactory DCB angioplasty. After DCB angioplasty, if there is a deterioration of blood flow (TIMI grade flow ≤ 2) after DCB angioplasty, it is recommended to give intracoronary medication (e.g., nitroprusside) and wait approximately 5 min before making the final assessment. In cases when subjects experience dissection type D, E, and F (NHLBI classification) or visual residual stenosis > 30%, a second-generation sirolimus-eluting stent, which is the same stenting used in the Reference arm, will be implanted mandatory as the bailout stent. These participants will be considered as a part of the primary DCB angioplasty strategy and included in the primary analyses.

The device used for primary DCB angioplasty is the Swide DCB (Shenqi Medical, Shanghai, China), which is a balloon coated with a paclitaxel-iopromide formulation (3 µg Paclitaxel per 1 mm^2^ of the balloon surface) using a proprietary dipping process that deposited the formulation preferentially in the folds of the balloon [20]. The spray coating of the mixture of paclitaxel and iopromide of the DCB is via ultrasound, with the crystal size < 2 μm. Previously, the Swide DCB has demonstrated non-inferiority to the SeQuent Please DCB, which is also a paclitaxel-iopromide coated DCB (3 µg Paclitaxel per 1 mm^2^), for the primary endpoint of 9-month in-segmentlate loss in patients with in-stent restenosis [21].

#### Randomization to the primary stenting strategy

The performance of primary stenting adheres to the routine local clinical practice and established guidelines [2, 15, 16]. In the primary stenting group, the Firebird 2 DES will be used. The Firebird2 DES (MicroPort, Shanghai, China) is a sirolimus-eluting coronary stent with an L605 Co-Cr alloy platform and durable polymer. The strut is 86 μm in thickness, and 80% of the drug is released within 30 days. The effectiveness and safety of the Firebird2 DES have been confirmed in a real-world population and randomized cohorts [22–24]. If delivery failure occurs, an alternative stent may be utilized [25].

### Concomitant medication and treatment

All study patients are administered antithrombotic drugs according to international guidelines [26]. All subjects must receive DAPT, being aspirin and either clopidogrel or ticagrelor for at least one month after index PCI, followed by aspirin, clopidogrel, or ticagrelor monotherapy indefinitely. Detailed recommendations for pre-procedural and post-procedural antiplatelet regimens are shown in Supplementary Table 1. While the physician has discretion over other medical treatments, it is strongly advised to implement guideline-directed medical therapy to address the patient’s specific condition, such as controlling hypertension or diabetes mellitus, prescribing high-intensity statins, discontinuing cigarette smoking, and providing optimal pharmacologic treatment for heart failure. All antiplatelet medications (including start and stop times of interrupted DAPT) and other cardiac medications will be recorded in the eCRF at each visit.

### Follow-up

Scheduled follow-up visits occur at 1 (± 14 days), 3, 6, 12, 18, and 24 (± 30 days) months post-randomization. After 24 months, the follow-up will be conducted annually and kept for up to 10 years. All follow-up visits are preferably scheduled on-site. If the patients are unable or unwilling to visit the outpatient clinic, the scheduled visit can be replaced by a telephone call except for the 30-day, 1- and 2-year visits. At each visit, self-reported adherence to the prescribed medications is collected with the assessment of any cardiac or cerebrovascular ischemic or bleeding occurrences or any serious adverse event. Each participant’s WeChat account will be documented for record-keeping purposes. To facilitate the acquisition of patient-reported outcomes and adherence to the prescribed medications, we developed a mobile application that functions through the WeChat platform. All participants will be contacted monthly through this application and receive a questionnaire to evaluate their health status and adherence.

### Study endpoints

The study endpoints are listed in Table 2. The primary endpoint is the Device-oriented Composite Endpoint (DoCE) within 24 months after randomization. DoCE is defined as a composite clinical endpoint of cardiovascular death, target vessel myocardial infarction (TV-MI), and clinically indicated target lesion revascularization (CPI-TLR) [27]. The definition of Academic Research Consortium (ARC)-2 will be followed [27]. Cardiovascular death is defined as any death due to a cardiac cause, unwitnessed death, death of unknown cause, and all study procedure-related deaths [27]. MI will be defined using the SCAI consensus for peri-procedure MI within 48 h of the index procedure [28], and the Fourth Universal Definition of MI > 48 h after the index procedure [29]. TV-MI is defined as MI that cannot clearly be attributable to a non-target vessel. TLR is defined as a repeat percutaneous intervention of the target lesion or bypass surgery of the target vessel performed for restenosis or other complications of the target lesion. Clinically and physiologically indicated TLR will be adjudicated based on the assessment of a positive functional ischemia test by either Wire-based or angiographic-derived Fractional Flow Reserve or Quantitative Coronary Analysis, with explicit criteria provided in the Supplementary Methods. The definition of device and procedure success are also provided in the Supplementary Methods. For secondary endpoints, stroke is defined as any non-convulsive focal or global neurological deficit of abrupt onset lasting for more than 24 h or leading to death, which is caused by ischemia or hemorrhage within the brain. The Neuro-ARC definition and classification will be used [30]. Bleeding will be defined by the Bleeding Academic Research Consortium (BARC) criteria [31], and other definitions [32–36] will used for exploratory purposes. The adherence to the medication will be assessed according to the Non-adherence Academic Research Consortium (NARC) [37] definitions.

**Table 2:** Study Endpoints

**Primary endpoint** Device-oriented Composite Endpoint (DoCE), defined as a nonhierarchical composite clinical endpoint of cardiac death, target vessel myocardial infraction (TV-MI), and clinically and physiologically indicated target lesion revascularization (CPI-TLR) (Time Frame: 24 months)	
**Secondary endpoints**	
1. DoCE (Time Frame: 1, 12, 36 and 60 months)	
2. Individual components of the DoCE (Time Frame: 1, 12, 24, 36 and 60 months)	
3. Patient-oriented composite endpoint (PoCE) (Time Frame: 1, 12, 24, 36 and 60 months) *PoCE is a nonhierarchical composite clinical endpoint of all-cause death, any stroke, any MI, and any revascularization*	
4. Individual components of the PoCE (Time Frame: 1, 12, 24, 36 and 60 months)	
5. Target vessel failure (TVF) *Target vessel failure is a nonhierarchical composite clinical endpoint of cardiac death, TV-MI, and clinically and physiologically indicated target vessel revascularization (CPI-TVR)*	
6. Clinically and physiologically indicated target vessel revascularization (Time Frame: 1, 12, 24, 36 and 60 months)	
7. Net adverse clinical events (NACE) (Time Frame: 1, 12, 24, 36 and 60 months) *NACE is a nonhierarchical composite clinical endpoint of all-cause death, any stroke, any MI, any revascularization, and BARC-defined type 3 or 5 bleeding events*	
8. Definite/Probable Stent thrombosis rates according to ARC-II classification (Time Frame: 1, 12, 24, 36 and 60 months)	
9. BARC type 3 or 5 bleeding events (Time Frame: 1, 12, 24, 36 and 60 months)	
10. BARC type 2, 3 or 5 bleeding events (Time Frame: 1, 12, 24, 36 and 60 months)	
11. BARC defined type 2 bleeding events (Time Frame: 1, 12, 24, 36 and 60 months)	
12. Device success rate (Time Frame: Post-procedure)	
13. Procedure success rate (Time Frame: 7 days post-procedure)	
14. Hierarchical composite clinical endpoint of cardiac death, TV-MI, and CPI-TLR (Time Frame: 1, 12, 24, 36 and 60 months)	
15. Hierarchical composite clinical endpoint of any death, any stroke, any MI, BARC defined type 3 bleeding events, any revascularization and BARC type 2 bleeding events (Time Frame: 1, 12, 24, 36 and 60 months)	

Suspected adverse events will be reported promptly on an electronic case report form, with source documents centrally collected. After collecting adverse events centrally, any record that could lead to the unblinding of treatment assignment will be obliterated before submission to the clinical event committee (CEC). All adverse events will be categorized according to predefined criteria by an independent clinical-event adjudication committee whose members are unaware of the assignment group. However, if the CEC members reviewed the angiogram, due to the absence of a metallic scaffold in the primary DCB group (unless the patient had bailout stenting), the blinding of the assignment group might not be feasible.

### Off-line quantitative coronary angiographic measurements (QCA) and quantitative flow ratio (QFR) measurement

The off-line quantitative coronary angiography (QCA) [38] and quantitative flow ratio (QFR) [39] assessment by an independent Corelab will be performed at baseline (pre- and post-PCI). Routine follow-up angiography in the absence of symptoms was not recommended. For the purpose of adjudicating clinically indicated or physiologically indicated revascularization, QCA, and QFR measurements will be conducted if the angiogram of revascularization is assessable.

### Sample size calculation

This study compares treatment groups at the individual patient level. Our hypothesis is that for the treatment of *de novo*, non-complex lesions, the primary DCB angioplasty group would be non-inferior to the primary stenting group in terms of Device-oriented composite endpoint within 24 months after PCI.

Due to the limited availability of dedicated data on the occurrence rate of DoCE at two years in patients of non-complex lesions, the event rate of the primary stenting group in this trial was estimated by referring to the findings of the GLOBAL LEADERS subgroup analysis of complex/non-complex PCI [17], in which the patients with non-complex PCI had 2-year cumulative event rate of DoCE of 6.7%, and the findings of the contemporary all-comers DES trials, including TARGET AC [40], TALENT [41], DESSOLVE III [42], BIONYX trials [43], in which patients had 2-year cumulative event rate of DoCE ranging from 6.9 to 8.7%. It was assumed that patients treated with different second-generation DES would have a similar cumulative event rate of DoCE. Therefore, it is anticipated that 6.7% of patients in the primary stenting group will reach the primary endpoint of DoCE at two years. The non-inferiority margin of 2.68%, which was 40% of the cumulative event rate of DoCE, was chosen based on clinically acceptable relevance according to the margins in previous major trials of comparing DCB to DES [44], or one DES comparing to another DES [40–43], and the feasibility of patient enrolment. With a total of 2156 patients (1078 per group), the study is estimated to have 80% power to show non-inferiority with a 5% one-sided α error rate [3, 40–43]. Accounting for an attrition rate of approximately 5%, the final sample size was determined to be at least 2270 patients (1135 per group).

### Statistical considerations

The demographic and clinical variables at baseline will be summarized for each treatment group, considering the intention-to-treat (ITT), per-protocol, and as-treated populations. Categorical data will be described as numbers (percentages). Continuous variables will be expressed as mean ± standard deviation or median (interquartile range) for normal or skewed distributions.

The primary endpoint of the trial is DoCE at 24 months after randomization. The primary analysis will be performed based on a crude measurement of treatment difference between groups in the primary endpoint, without adjusting for any covariates, using the intention-to-treat (ITT) population. To estimate the cumulative event rate of DoCE at 24 months in each group, the Kaplan-Meier (KM) method will be employed. The one-sided 95% confidence interval (CI) for the difference in the cumulative event rate at 24 months between the primary DCB angioplasty group and the primary stenting group will be calculated using Greenwood’s formula for the variance of the KM estimates. If the upper limit of the one-sided 95%CI is below 2.68%, it will be concluded that the primary DCB angioplasty group is non-inferior to the primary stenting group. If the non-inferiority testing for the primary endpoint is met, the superiority testing of the primary endpoint will be further tested.

In addition, a covariate-adjusted analysis of the primary endpoint using the inverse probability of treatment weighting approach, considering the covariates at baseline and center effect, will be performed in the ITT population as a sensitivity analysis. The crude and adjusted analyses will be repeated in the per-protocol and as-treated population to support the primary results. For secondary endpoints, the difference in cumulative event rate and their two-sided 95%CIs will be reported, and Cox proportional hazard ratios (HR) will also be provided.

The prespecified subgroup analyses will also be conducted for clinically relevant factors such as age, sex, body mass index, diabetes mellitus or smoking, and other risk indicators, with details described in Supplementary Table 2. Stratum-specific HRs and corresponding 95% CI will be calculated for each subgroup using a Cox proportional hazards model. Interaction testing will be performed using the subgroup X treatment as an additional term in the Cox model. A prespecified landmark analysis of the primary endpoint will be performed from 0 to 12 and 12 to 24 months. Unless otherwise specified, a two-sided test will be utilized for testing at a 5% significance level. All analyses will be described in detail in the statistical analysis plan, which will be developed and finalized before the database lockup.

### Safety monitoring

The Data and Safety Monitoring Board (DSMB), in conjunction with the steering committee responsible for ensuring participant safety, will act in an advisory capacity to monitor participant safety, evaluate the study progress, and review procedures for maintaining data confidentiality. A biannual DSMB meeting will be held, either in-person or via teleconference, to discuss study progress, ensure proper execution of study procedures, maintain data quality and security, and review any safety concerns related to participants. Although no interim analysis was initially planned, the DSMB holds the power to terminate the study process and scrutinize relevant events during the trial in the event of any safety issues.

## Discussion

In 1996, the US Food and Drug Administration approved the first 2 bare-metal stents (BMS) for the treatment of de novo lesions to prevent recoil and for treatment of acute artery closure after plain old balloon angioplasty (POBA) [45]. Meanwhile, pivotal randomized trials compared BMS with POBA, and the results indicated that the use of BMS led to a reduction of adverse events by 30% within the first six months after PCI. This reduction was primarily attributed to a 50% decrease in the risk of repeat revascularization [45, 46]. Furthermore, these trials established an important concept: stents could effectively decrease restenosis by providing significant initial angiographic gain and by preventing early recoil and late negative remodeling of the treated vessel [47, 48].

However, there was concern that primary stenting without first trying to obtain satisfactory results with POBA alone would increase the occurrence of in-stent restenosis. Consequently, in 2000, studies were conducted to compare primary stenting using BMS versus POBA with the provisional use of BMS [49–51]. These trials revealed several common findings, one of which was the difficulty in attaining a satisfactory result solely with POBA. Using angiographic criteria alone, the OCBAS trial [49] showed that 13.5% of the POBA group crossed over to stent implantation. When both angiographic and physiologic criteria were employed to determine an optimal outcome, up to 50% of patients failed to achieve a satisfactory result. Furthermore, the clinical outcome with primary stenting is as good or better than that achieved with a strategy of provisional stenting [45].

After the mechanical era in interventional cardiology, as represented by POBA and BMS, the local dispensing era emerged with the delivery of anti-restenotic drugs directly into the coronary artery [52]. The introduction of DES revolutionized the field and established itself as the preferred treatment for patients undergoing PCI [2]. However, the risk of late stent thrombosis and restenosis after DES is still approximately 2% per year and leads to a target-lesion failure rate of approximately 14% after 5 years [2, 53].

DCBs were initially introduced in the European market in 2007, with the aim of being an alternative strategy for ISR instead of DES [2, 54]. The rationale behind the utilization of DCB is founded on the concept that highly lipophilic drugs can achieve effective drug delivery even with short contact durations between the balloon surface and the vessel wall, thus theoretically avoiding the side effects associated with the maladaptive biological response induced by permanent prosthesis implantation [2]. Based on these theoretical foundations, DCB has demonstrated a correlation with faster vascular healing, a favorable effect on preventing late negative remodeling, and even provides late lumen enlargement [55, 56].

Two decades after the early trials comparing primary stenting with BMS vs. POBA with provisional stenting, in the era of DES and DCB, together with the introduction of newer balloons such as cutting and scoring balloons for achieving satisfactory angiographic results, DCB angioplasty with provisional stenting has been explored for the treatment of *de novo* coronary lesions in some specific settings, demonstrating notable advantages, particularly in SVD [57, 58]. The BASKET-SMALL 2 trial [6, 9], which enrolled 758 participants randomly allocated to DES or DCB, is the largest study to date investigating *de novo* SVD. The study demonstrated that DCB angioplasty with provisional DES implantation was non-inferior to primary DES in terms of major adverse cardiovascular events over a period of 3 years. Similarly, the PICCOLETO II trial, which also focused on patients with SVD, found no significant difference in clinical outcomes at 12 months between the DCB and DES arms. However, the DCB arm showed significantly lower late lumen loss (LLL) than the DES arm [59]. Furthermore, DCBs have also exhibited potential advantages in other *de novo* settings, including patients with a higher risk of bleeding or those encountering high thrombus burden and inflammatory states [11, 12].

However, there remains a scarcity of randomized data that compares the clinical outcomes associated with the use of DCB angioplasty and DES in the context of *de novo* disease with all vessel diameters. The available findings show significant variability, with most of the existing data coming from small-scale angiographic investigations or clinical follow-ups that solely focus on the DCB arm [19]. The findings of the REVELATION trial [11] demonstrated that DCB was comparable to DES in terms of the primary endpoint, fractional flow reserve (FFR), during a 9-month angiographic follow-up of 120 patients who underwent primary PCI for ST-elevation myocardial infarction. The trial also revealed similar angiographic late lumen loss (LLL) and clinical outcomes between the two treatment modalities. Nishiyama et al. [60] have also reported no significant difference in MLD or late lumen loss between the DES and DCB in large vessels. Conversely, there are also studies [61] showing a higher LLL in patients with acute coronary syndrome when treated with DCB compared to DES.

Considering the limitations of stents and the basis of promising evidence indicating the effectiveness of DCB angioplasty in treating ISR and SVD, there is potential for primary DCB with provisional stenting to serve as a viable substitute for primary DES implantation in treating *de novo* lesions across all vessel diameters. To validate this hypothesis, we designed the REC-CAGEFREE I study, which aimed to enroll a large cohort to investigate the non-inferiority of primary DCB angioplasty to primary stenting in patients with *de novo* lesions without any limitations on vessel diameter.

### Current status of the REC-CAGEFREE I trial

The REC-CAGEFREE I trial commenced with the enrollment of the first patient in February 2021 and concluded with the enrollment of the last patient in May 2022 (Supplementary Fig. 1). A total of 2,272 patients were ultimately enrolled from 43 participating sites. Follow-up for the primary endpoint will be finalized in June 2024, and all the participants will be monitored for up to 10 years after randomization. The findings from the primary analyses are anticipated to be published in the third quarter of 2024.

## Conclusion

The REC-CAGEFREE I trial is the first randomized trial with a large cohort and clinical endpoint to assess the non-inferiority of primary DCB angioplasty to primary stenting to treat *de novo*, non-complex lesions in all vessel diameters. If non-inferiority is shown, PCI with primary DCB angioplasty could provide as an alternative treatment option to primary stenting.

### Electronic supplementary material

Below is the link to the electronic supplementary material.

Supplementary Material 1

## Data Availability

The datasets used and/or analysed during the current study available from the corresponding author on reasonable request.
