# Supplementary material for: Drug-coated balloon angioplasty with provisional stenting versus primary stenting for the treatment of de novo coronary artery lesions: REC-CAGEFREE I trial rationale and design
Source: BMC Cardiovasc Disord. 2024 Jun 24;24:319. doi: 10.1186/s12872-024-03974-0 (PMC11194892; doi:10.1186/s12872-024-03974-0)

Supplemental material

**Primary drug-coated balloon angioplasty with provisional stenting versus primary stenting for the treatment of de novo coronary artery lesions: REC-CAGEFREE I trial rationale and design**

This supplementary material has been provided by the authors to give readers additional information about their work.

**Supplementary Methods**

**Study endpoints**

**Clinically and** **physiologically indicated revascularization (CPI-TLR/TVR)**

A revascularization is considered clinically and physiologically indicated if associated with any of the following (Hierarchically):

1. Positive invasive functional ischemia test (e.g. FFR, iFR). When invasive functional assessment is available, use the following hierarchy:
   1. Core laboratory–reported fractional flow reserve ≤0.80 or instant wave-free ratio ≤0.89.
   2. Site-reported fractional flow reserve ≤0.80 or instant wave-free ratio ≤0.89.
   3. Core laboratory–reported angiography-derived fractional flow reserve ≤0.80.
2. Angiographic diameter stenosis ≥50% (by core laboratory QCA based on the average of multiple views) and positive non-invasive ischemia test (e.g. dobutamine stress test, nuclear test, exercise test, FFR-CT).
3. Angiographic diameter stenosis ≥50% (by core laboratory QCA based on the average of multiple views) and ischemic symptoms (stable angina or acute coronary syndrome).
4. Angiographic diameter stenosis ≥70% (by core laboratory QCA based on the average of multiple views).
5. Angiographic diameter stenosis ≥70% (by core laboratory QCA based on the worst view).

Additional notes:

- In case a revascularization occurs in a vessel and the core laboratory-reported angiography-derived fractional flow reserve > 0.80, and the target lesions have a core laboratory-reported angiographic diameter stenosis ≥70% by QCA based on multiple views, the revascularization will be considered as non-clinically indicated.
- When the diameter stenosis is <50%, the presence of severe ischemic signs and symptoms (e.g., acute myocardial infarction) would also confirm the diagnosis of a clinically indicated revascularization.

**Device success**

**Device success of DCB (Lesion Based)**

Device Success of DCB is defined by fulfilling all the following conditions:

1. Successful delivery within 120 seconds (DCB in the vessel) of the DCB device at the intended target lesion (Multiple attempts with the same DCB are allowed. If unable to deliver the DCB within 120 seconds or required a new DCB, then it is considered as device failure).

2. DCB is successfully dilatated for at least 30 seconds, and the device system is successfully withdrawn.

3. After DCB dilatation, the target vessel has no flow-limiting dissection (type D, E, and F), and the final in-lesion residual stenosis is less than 30% by core laboratory QCA (preferred methodology) or visual assessment.

4. No bailout procedure by the stent.

**Device success of DES (Lesion Based)**

Device Success of DES is defined by fulfilling all the following conditions:

1. Successful delivery, balloon expansion, and deployment of the assigned device at the intended target lesion (Multiple attempts using the same DES are allowed; for example, success at a second attempt with the same DES after rewiring the vessel, use of a support catheter, or additional ballooning, vessel preparation, etc)

2. Successful withdrawal of the device delivery system.

3. Attainment of a final in-stent residual stenosis of <20% by core laboratory QCA (preferred methodology) or visual assessment.

Additional notes:

- Using a second (or more) assigned device(s) or non-assigned devices due to failure of the first assigned device is classified as device failure for the target lesion.
- When deployment of more than one assigned device is planned for a single target lesion (e.g., overlapping devices for a long lesion or a two-stent strategy for a bifurcation lesion), all assigned devices are assessed and reported as one device. In that case, only when all assigned devices are successfully implanted at the intended target lesion is this classified as device success.
- Using bailout devices (as allocated by randomization) due to edge dissections or geographic misses is not regarded as a device failure but rather as a clinical issue.
- Successful deployment includes the expansion of the delivery balloon to its appropriate diameter, as indicated on the balloon compliance chart.
- Deployment failure is classified as device failure. It is independent of whether the delivery device was safely removed or not (a failure removal of the delivery device or removal concomitated with procedural complication will be documented separately).

**Procedure success**

**Procedure success of DCB (Patient Based)**

Procedure success of DCB is defined as Device success of DCB + without the occurrence of DoCE + no stent thrombosis at discharge during the index procedure hospital stay (maximum of 7 days).

**Procedure success of DES (Patient Based)**

Procedure success of DES is defined as Device success of DES + without the occurrence of DoCE + no stent thrombosis at discharge during the index procedure hospital stay (maximum of 7 days).

**Supplementary Table 1 Antiplatelet therapy regimen**

|  | **Pre-procedural** | **Post-procedural** |
| --- | --- | --- |
|  | It is recommended that a loading dose of aspirin and a P2Y12 inhibitor be administered from 0 hours to 24 hours before the procedure. | All subjects must receive dual antiplatelet therapy (DAPT), aspirin, and a P2Y12 inhibitor for at least 1 month after PCI.  According to standard local practice, DAPT can be de-escalated or abbreviated after 1 month by Aspirin, Clopidogrel, or Ticagrelor monotherapy. Extended DAPT will be at the discretion of the investigator. |
| Aspirin | Any subject not already taking daily chronic aspirin therapy will receive a loading dose of 300 mg | A maintenance dose of 100 mg QD |
| **P2Y12 inhibitors** | |  |
| Clopidogrel | A peri-procedural loading dose of 600 mg is recommended (for subjects already on chronic clopidogrel therapy [75 mg, ≥7 days], a loading dose of 300 mg may be administered) | A maintenance dose of 75 mg QD |
| Ticagrelor | A peri-procedural loading dose of 180 mg is recommended | A maintenance dose of 90 mg BID |

DAPT: dual-antiplatelet therapy; PCI: percutaneous coronary intervention.

**Supplementary Table 2 Subgroup analyses**

Concomitant medication and risk factors at baseline:

1. Treatment in relation to age at the time of randomization (<75 or ≥75)
2. Treatment in relation to sex (male, female)
3. Treatment in relation to BMI at the time of randomization (<25 kg/m^2^, ≥25 kg/m^2^)
4. Treatment in relation to hypertension status at the time of randomization (yes, no)
5. Treatment in relation to hyperlipidemia status at the time of randomization (yes, no)
6. Treatment in relation to diabetes status at the time of randomization (yes, no)
7. Treatment in relation to renal function at the time of randomization (yes, no)
8. Treatment in relation to smoking status (yes, no)
9. Treatment in relation to COPD at the time of randomization (yes, no)
10. Treatment in relation to PVD at the time of randomization (yes, no)
11. Treatment in relation to the history of cardiovascular disease (yes, no)
12. Treatment in relation to the history of stroke or TIA (yes, no)
13. Treatment in relation to LVEF at the time of randomization (≥50%, <50%)
14. Treatment in relation to baseline WBC level (<median, ≥ median)
15. Treatment in relation to the history of CHF (yes, no)
16. Treatment in relation to heart rate at the time of randomization (<median, ≥ median)
17. Treatment in relation to high bleeding risk according to ARC definition at the time of randomization (yes, no)
18. Impact of bleeding scores (PRECISE-DAPT, DAPT, CRUSADE, ACUITY, PARIS score) in risk stratifying bleeding and ischaemic events and its interaction with treatment
19. Impact of SYNTAX score and its derived scores (functional SYNTAX score, SYNTAX Score II, residual SYNTAX score, logistic clinical SYNTAX Score) in risk stratifying bleeding and ischaemic events and its interaction with treatment

Clinical presentation

1. Treatment in relation to the clinical presentation at the time of randomization (STEMI, NSTEMI, UA, CCS)

PCI procedure

1. Association of non-compliant balloons versus cutting or scoring balloons regarding pre-dilatation successful rates (yes, no)
2. Association of unsuccessful pre-dilatation versus successful pre-dilatation with prognosis (yes, no)
3. Treatment in relation to multi-vessel disease (1-vessel disease, 2-vessel disease)
4. Treatment in relation to bifurcation (yes, no)
5. Impact of angiography-derived fractional flow reserve/angiography-derived index of microcirculatory resistance pre- and post-PCI on ischemic events
6. Treatment in relation to lesion location (LAD, RCA, LCX)
7. Treatment in relation to proximal LAD (yes, no)
8. Treatment in relation to the device to lesion length (≥28 mm, <28mm)
9. Treatment in relation to center and operator experience (≤median, >median)
10. Treatment in relation to staged PCI (yes, no)
11. Impact of DS% pre- and post-PCI
12. Impact of QFR/FFR pre- and post-PCI
13. Treatment in relation to device diameter (< 3.0mm, ≥3.0mm)

Medication

1. Impact of adherence to antiplatelet therapy (yes, no)
2. Optimal medical treatment (OMT) specified in the guidelines (yes, no)
3. Statin/PCSK-9 inhibitor (yes, no)

COVID-19

1. Impact of COVID-19

BMI, body mass index; COPD, chronic obstructive pulmonary disease; PVD, peripheral vascular disease; TIA, transient ischemic attack; LVEF, left ventricular ejection fraction; ARC, Academic Research Consortium; STEMI, ST-elevation myocardial infarction; NSTEMI, non-ST-elevation myocardial infarction; UA, unstable angina; CCS, chronic coronary syndrome; LAD, left anterior descending coronary artery; RCA, right coronary artery; LCX, left circumflex coronary artery.

**Supplementary Figure 1** **Enrollment progress**


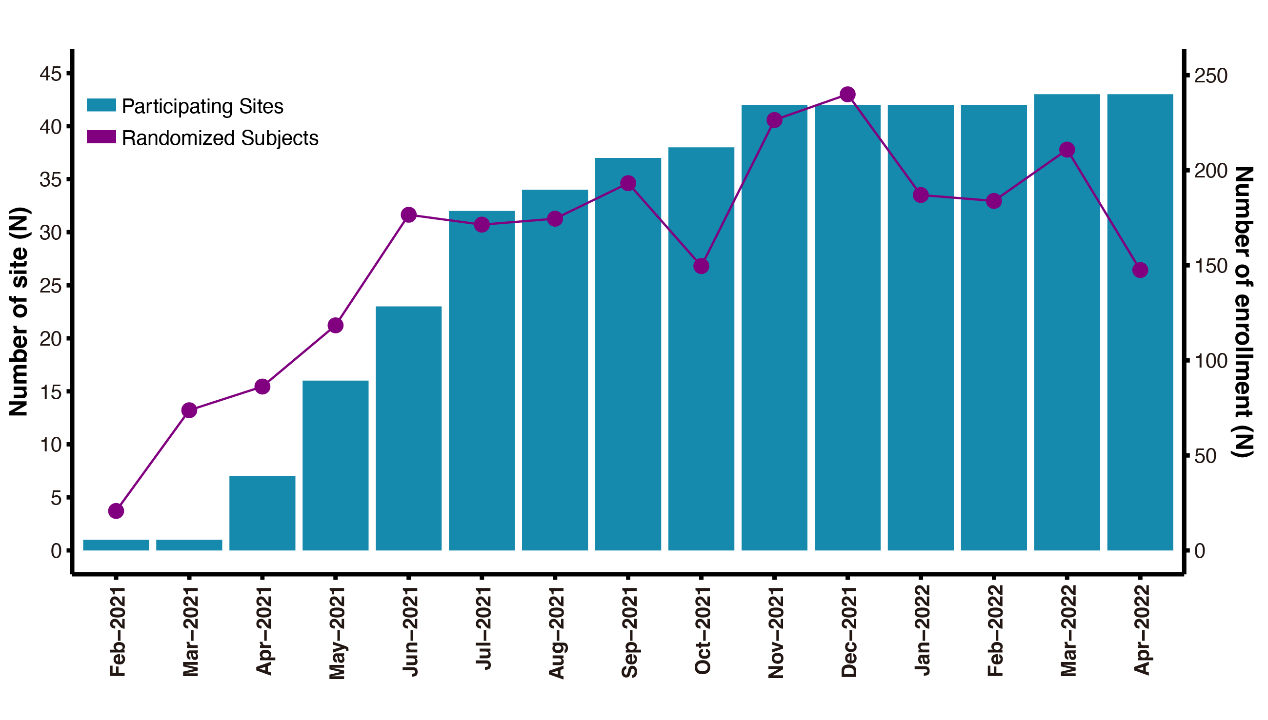

Supplement: Supplementary file 1 — Supplementary Material 1 [file 12872_2024_3974_MOESM1_ESM.docx]
